# How a steeper organisational hierarchy prevents change—adoption and implementation of a sustainable employability intervention for employees in low-skilled jobs: a qualitative study

**DOI:** 10.1186/s12889-022-14754-w

**Published:** 2022-12-17

**Authors:** Emmelie Hazelzet, Inge Houkes, Hans Bosma, Angelique de Rijk

**Affiliations:** Department of Social Medicine, Faculty of Health, Medicine and Life Sciences, CAPHRI Care and Public Health Research Institute, Maastricht University, 6200 MD Maastricht, The Netherlands

**Keywords:** Adoption, Organisational hierarchy, Implementation, Intervention, Power, Sustainable employability

## Abstract

**Background:**

Adoption and implementation are prerequisites for the effectiveness of organisational interventions, but successful implementation is not self-evident. This article provides insights into the implementation of the organisational intervention ‘Healthy Human Resources’ (HHR). HHR is developed with Intervention Mapping and aims at improving sustainable employability (SE) of employees in low-skilled jobs.

**Methods:**

Qualitative data on adoption and implementation were collected by interviews with three employees and seven middle managers in five Dutch organisations and by extensive notes of observations and conversations in a logbook. Data triangulation was applied and all data were transcribed and analysed thematically using the qualitative analysis guide of Leuven (QUAGOL).

**Results:**

All organisations adopted HHR, but three failed during the transition from adoption to implementation, and two implemented HHR only partially. The steepness of the organisational hierarchy emerged as an overarching barrier: steeper hierarchical organisations faced more difficulties with implementing HHR than flatter ones. This was reflected in middle managers’ lack of decision-making authority and being overruled by senior management. Middle managers felt incapable of remedying the lack of employees’ voice. Subsequently, ‘us-versus-them’ thinking patterns emerged. These power imbalances and ‘us-versus-them’ thinking reinforced each other, further strengthening the hierarchical steepness. Both processes could be the result of wider socio-political forces.

**Conclusions:**

This study improved the understanding of the difficulties to adopt and implement such organisational intervention to contribute to the sustainable employability of employees in low-skilled jobs. Practical implications are given for future implementation of organisational interventions.

## Background

More and more organisations are implementing organisational interventions to contribute to their employees’ health [1–4]. Employers often rely on ready-made health programmes from third parties (often commercial) and face challenges to implement them successfully, often because of a top-down implementation. Academics also face the challenge of developing scientifically and practically relevant interventions to promote employees’ health or sustainable employability (SE) [5, 6]. A particularly difficult and at the same time vulnerable group in this regard consists of employees in low-skilled jobs [1, 7, 8]. These employees have significantly higher risks of poor health and more adverse work conditions compared to employees in higher-skilled jobs [9, 10]. This group barely participates in organisational health interventions, presumably due to a mismatch between these interventions and their specific needs [8]. However, organisational interventions may potentially be effective to reduce health inequalities among employees at the workplace [11].

To address these challenges and to improve the SE of employees in low-skilled jobs, the organisational intervention ‘Healthy Human Resources’ (HHR) was developed in close cooperation with employees and employer representatives following the Intervention Mapping approach [12]. This approach is widely used for the development of tailored, theory- and evidence-based programs suited to the needs of a specific population and strongly built on stakeholder involvement. HHR is a web-based step-by-step toolkit to support joint groups of middle and human resource (HR) managers and employees in low-skilled jobs to develop and implement SE interventions tailored to their organisation and needs, via a dialogue-based participatory approach. HHR consists of seven steps, each represented by tasks and supportive dialogue-based tools (e.g., brainstorming working formats) for performing the tasks. More details about the content and the theoretical development of HHR have been reported elsewhere [13, 14]. HHR stimulates middle managers (the HHR-user) to involve their employees actively from the beginning of this process. This allows employees to have more voice and contributes to a more egalitarian and collective decision-making process, which is expected to improve their SE. Five organisations participated in the development of HHR and started to adopt and implement it.

In this article, adoption refers to the decision to use the intervention, while implementation refers to the actual usage of the intervention in daily practice [15]. The theoretical framework of Fleuren et al. [16] suggests that the adoption and implementation processes can be affected (positively or negatively) by factors at four levels: (1) the socio-political context level (e.g. external forces, societal and political structures and developments); (2) the organisation level (e.g. organisational culture and lack of available resources); (3) the user level (e.g. lack of positive attitude, motivation, perceived social support); and (4) the intervention level (e.g. lack of compatibility and alignment with the organisation) [17]. In this article, two types of users are distinguished: the employees targeted by HHR (end user) and the middle managers, the main user of HHR (intermediate user), whose actions determine the degree of exposure of the employees to HHR [15].

The aforementioned barriers in the theoretical framework of Fleuren et al. are reported for organisational, health-focused interventions and often result in implementation failure [18–22]. They are expected to be even more pronounced in organisations with low-skilled jobs. These employees often experience high job demands and low job control associated with several negative health effects [11, 23, 24]. They often perform simple and routine work tasks, which is more common in more hierarchical, centralised organisations [25].

However, the distinction of these four levels seems insufficient to fully understand the process of adoption and implementation. Previous studies in the area of occupational health pointed at adoption-implementation gaps and underscored the complex, dependent nature of both phases [1, 26]. This article aims first to study the degree of adoption and implementation of the organisational intervention HHR in a sample of various organisations, and next, to understand the variation in these degrees across these phases. The research questions were: What was the degree of adoption and implementation of HHR in various organisations, and how can the variation in adoption and implementation in these organisations be understood? A better understanding will contribute to improving the future implementation of new organisational interventions focusing on occupational health [18, 27–30], particularly those with a participatory approach at work [31].

## Methods

### Study design

This qualitative study used an explorative and retrospective design based on thematic analyses of logbook entries, observations and interviews collected between September 2018 and September 2020 in five Dutch organisations. This study design used data triangulation to obtain a complete and holistic understanding of the adoption and implementation processes from multiple stakeholder perspectives. Ethical approval was obtained from the Medical Ethical Committee of the academic hospital in Maastricht, The Netherlands (METC 2017–0311). Employers and employees of the participating organisations signed an informed consent form prior to their participation. The COnsolidated criteria for REporting Qualitative research (COREQ) checklist were followed [32] to ensure the quality of reporting methods and results.

### Organisational settings and sample

Five Dutch organisationsdeploying employees in low-skilled jobs were recruited via the network that was established by the researchers in an earlier study for the development of HHR [14]. The five organisations participating in this study were: 1) a governmental institution, 2) a cleaning company, with different worksites, 3) a warehouse, 4) a manufacturing company, and 5) a meat-processing company. The organisations were purposively selected by focusing on low-skilled jobs in diverse sectors. The sizes of these organisations varied, ranging from 40 to almost 4000 employees. In four of the five organisations, the employees mainly performed physically demanding work, while the employees in organisation 1 performed relatively simple administrative tasks (deskwork). Employer representatives were defined as professionals in the organisation who initiated HHR (i.e. HR managers, line managers and reintegration advisors, hereafter: middle managers). Within the Dutch context, employers are responsible for sickness absence prevention and management [33]. In larger organisations, specific professionals are employed to address sickness absence (and its prevention) and facilitate this process (e.g. reintegration advisor) and were therefore included in this study sample. The middle managers were the first contact persons for the researchers in the earlier (development) study and a relationship already existed between these managers and the researchers (EH and IH). With respect to the interviews, the researchers purposively selected seven middle managers who were approached via phone or email. In addition, employees were approached by their employer, and participated voluntarily. Inclusion criteria for the interviewees were: at least one employer representative of each organisation, such as middle managers, who initiated and were familiar with HHR and 2) employees who performed low-skilled work, mostly with a lower level of education and speak the Dutch language.

### Data collection

Data triangulation was applied by using the following data sources: logbook entries, observations and semi-structured interviews.

First, the number and content of all intervention contacts were tracked and documented in a logbook per organisation (in total five logbook entries). The intervention contacts were operationalised as an activity and consisted of both internal contact moments through various communication channels within the organisations (i.e. between middle managers and employees/senior management by email or meetings) and external contact moments (i.e. researchers and organisations by phone, email, on-site and online observations, and interviews). Events, materials shared and progress of the adoption and implementation of HHR within each organisation were also tracked.

Second, observations in terms of verbal and non-verbal expressions during external contact moments and through contextual observations during on-site visits were collected. Field notes during the on-site visits were documented. In total, 24 pages of observations were collected.

Third, semi-structured interviews were conducted by the researcher (EH) with seven middle managers and three employees of the organisations by telephone or online (based on the respondents' preferences) between June and September 2020. Respondents were familiar with EH from an earlier study on the development of HHR and knew the reasons for doing the research and the scientific background of EH. For practical reasons, two paired interviews took place (respondents 1 and 2 and respondents 5 and 6). Table 1 presents the characteristics of the respondents, who participated in the interviews.

**Table 1:** Respondents’ characteristics

Interview number	ID	Gender	Employment title	Organisation
1	1	Female	Reintegration advisor	Governmental institution (1)
	2	Female	Reintegration advisor	Governmental institution (1)
2	3	Female	HR manager	Cleaning company (2)
3	4	Male	Warehouse line manager	Warehouse (3)
4	5	Male	HR consultant	Manufacturing company (4)
	6	Female	HR consultant	Manufacturing company (4)
5	7	Female	HR manager	Meat-processing company (5)
6	8	Female	Employee	Warehouse (3)
7	9	Male	Employee	Warehouse (3)
8	10	Male	Employee	Warehouse (3)

A self-developed semi-structured interview guide with three main topics was used (Table 2). Topic 2 was included in the guide because the interviews took place during the COVID-19 pandemic, which might have affected the adoption and implementation. This interview format was self-developed to be consistent with the explorative design to collect information on specific circumstances that facilitated or hindered the degree of implementation within the various organisations. ‘On the spot’ member checking was performed by providing verbal summaries during and at the end of the interviews. Interviews lasted 39 min on average (range: 29–58 min) and were audio recorded. Data saturation was achieved by the interviews that took place after the other data had been collected. After the interviews, there was no opportunity to go back to the respondents for additional information due to time constraints and other priorities within the organisations. Since different data sources at different measurement moments were triangulated, cross-verification of the data was possible.

**Table 2:** Interview guide topics

**Topic 1**: Implementation of HHR
General experience HHR
Implementation of HHR (i.e. adoption process; experience of HHR-toolkit)
Barriers and facilitators of the implementation
**Topic 2**: Impact of COVID-19 on the adoption and implementation of HHR
General experience of COVID-19
**Topic 3**: Future implementation and continuation of HHR
Adaptations of HHR
Ideas about continuation of HHR

### Data analysis

Thematic analysis [34], the practical steps from the Qualitative Analysis Guide of Leuven (QUAGOL) [35] and the theoretical framework of the four levels of factors by Fleuren et al. (used as a lens to analyse the data) [16] formed the basis for data analysis. The data of the logbook and the interviews (audio-recorded and transcribed verbatim) were analysed simultaneously. The analysis process consisted of two parts: 1) the preparation of the coding process by paper and pencil work and 2) the actual coding process using qualitative software. Each part consisted of five stages. Table 3 summarises the stages of analysis. The stages in part 1 were conducted independently by two researchers (EH and AdR) and compared and evaluated by the other authors (IH and HB). During part 2, the actual coding process took place, using computer-assisted qualitative data analysis software, Nvivo program version 12. This part was performed by EH and continuously evaluated by AdR. During the final stages, the original data sources and narrative reports were regularly consulted to verify interpretation with all authors, and the data analysis was thus approached as an iterative process. Moreover, the degree of the adoption, transition and implementation was systematically determined. First, we returned to the performed data analyses and raw data and defined from the logbook the number of contact moments per organisation and categorised this per phase. Parallel, we checked the interviews and the field notes of the observations to see quotes/ expressions described supporting the phases. Based on this, together with all authors the degrees were classified into high, partial and low.

**Table 3:** Stages based on the Qualitative Analysis Guide of Leuven (QUAGOL)

**Part 1: Preparation of coding process**	
	Goal
Stage 1: Familiarisation—thorough (re) reading of the transcripts & logbook notes	A holistic understanding of the respondent’s experience – main message
Stage 2: Narrative report	Brief summary of the key storylines and essence of the interview and logbook notes
Stage 3: Translation of the narrative report into a conceptual scheme	The narrative report is translated into key concepts
Stage 4: Fitting test of the conceptual schemes	Create a dialogue of the conceptual schemes together within the research team to achieve optimisation
Stage 5: Constant comparison process	Forward–backward movement of comparison between within-case (one conceptual scheme per organisation) and across-case analysis (other conceptual schemes of other organisations)
**Part 2: Actual coding process**	
Stage 6: Drawing up a list of codes	Create a list of codes of the conceptual schemes without a specific order
Stage 7: Coding process	Link the relevant interview transcript fragments and logbook notes to an appropriate code
Stage 8: Analysis and description of concepts	Give a clear description of the concept, their meaning, dimension and characteristics
Stage 9: Extraction of the essential structure	Integration of all concepts in a meaningful conceptual framework Apply the four levels of Fleuren et al. to interpret the data
Stage 10: Description of the essential findings	

## Results

All organisations adopted HHR to varying degrees. These variations were amplified during the transition to implementation and the implementation itself. Different factors at various levels helped to understand this variation, but one overarching theme was found to understand impaired implementation: steepness of the organisational hierarchy. These three findings (1. degrees of adoption, transition and implementation; 2. Understanding adoption, transition and implementation; 3. Overarching theme:steepness of the organisational hierarchy) are addressed below in more detail.

### Degrees of adoption, transition and implementation

Degrees of adoption, transition and implementation varied across the five organisations. All organisations adopted HHR to some degree as expressed by the level of enthusiasm among the adopters (i.e. middle managers and senior management), ‘*Our production director who at the time fully endorsed it’ (ID: 5).* The adopters of organisations 1, 3 and 4 adopted HHR to the full extent, while the adopters in organisations 2 and 5 adopted the intervention to a limited extent, illustrating a lower level of enthusiasm. *‘I noticed that it took a lot of time, effort and energy so to say, to reach people, to mobilise people, to have them participate’ (ID:3).*

The degree to which the transition from adoption to implementation was made was low for organisations 2 and 5. Organisation 4 made many attempts (high number of contact moments [13]) to transition from adoption to implementation but eventually failed to continue the implementation. Only organisations 1 and 3 fully transitioned from adoption to implementation. The transition to implementation was characterised by enthusiasm together with the manager’s ability to translate HHR into concrete actions, *‘I’m positive about the project to this day, only it is just a difficult thing’ (ID:7).*

The implementation was characterised by enthusiasm, ability to take concrete actions and the actual use of HHR. Despite the enthusiastic middle managers and many attempts to continue, organisations 1 and 3 decided to stop during the implementation phase and failed to implement HHR to its full extent.

### Understanding adoption, transition and implementation

The three phases can be understood along with factors at the four levels [16] (Table 4). Strikingly, the user and intervention levels played a large role during adoption, while the organisation and socio-political context levels came into play more prominently towards and during implementation.

**Table 4:** Overview of factors per phase and level

Phase	**1) Adoption**	**2) Transition adoption -implementation**	**3) Implementation**
**Level**	**Overarching theme: steepness of the organisational hierarchy**		
**Socio-political context**		Occurrence of external shocks	Remaining shocks to the organisations
**Organisation**		Challenges faced in the workplace	Remaining shocks to the organisations
**Intermediate user (middle managers)**	The importance of support	The sandwich position Perception about employees – creating an in- & out-group	Appearance of mental fatigue
**End-user (employees)**		Perceptions of employees- ‘us- versus-them’ relationship	The feeling of not being taken seriously and a lack of communication
**Intervention HHR**	Alignment of HHR and organisational vision Positive impression about HHR	SE regarded as easy to embrace but difficult to implement	Pleasant way of working, but no guarantee for success

### Phase 1 Adoption of HHR

#### Intermediate user level – middle managers

##### The importance of support

The importance of support emerged during the adoption phase and was perceived both positively and negatively by the middle managers. Some interviewees experienced a broad support base from their senior management at the beginning of the adoption phase, but the support changed over time: *‘This was very much supported by the head office. We started with high hopes (…) it was highly prized and space was made available for it, they said: we will do that and people can participate in it and so on. (…) At one point, our HR director was fired, who considered sustainability very high (….) I see that happening very often, they say, We go back to basics ‘ (ID: 7).*

When no support was experienced, doubts arose and enthusiasm decreased. *‘The type of worker, the complexity of employees, spread over many locations, so we encountered a lot of problems with accessibility, how do we reach the right people? So how do you create support for the project?’ (ID: 3).* This lack of support seemed particularly disadvantageous for the employees. According to one manager, employees might not see the added value of HHR in combination with the observed organisational structure, which affected the employees’ support level. *‘Many people feel less connected to our organisation, so I don't think they're counting on it either’ (ID: 3).* Observations among employees in the cleaning company (organisation 2) confirmed this thought. They experienced a lack of connectedness with their employer and felt more connected to their host organisations (where they cleaned).

#### Intervention level

##### Alignment of HHR and organisational vision

When the HHR vision aligned with the company’s vision, adoption was perceived as easier. *‘That matched seamlessly with the strategic plan, seamlessly with everything’ (ID: 5).* Institutionalising HHR in the everyday core business processes was also regarded as important. The vision of HHR is regarded as ‘a way to act’ rather than as a separate project, which yields enthusiasm, a sign of adoption, in some organisations.

##### Positive impression about HHR

Interviewees clearly expressed positive attitudes towards HHR in the adoption phase. HHR was seen as a comprehensive and well-functioning toolkit. *‘You can call it a toolbox, clear steps, sequence, more like, I have a flyer here, I have a format here (…) Yes, I think it's neatly designed’ (ID: 4).* Additionally, HHR could help a HR manager to do a better job, but how to translate this to the workfloor and type of employee is difficult to imagine, because of the employees' profile and organisational structure: *‘HHR, a lot of solutions that you can use as an organisation for certain issues regarding health, sustainable employability. Not all of those solutions are feasible within our organisation and where I thought, well that fits, it’s also quite difficult to implement and to translate as a solution’ (ID: 3).*

### Phase 2 Transition adoption-implementation

The longer it took to transition from adoption to implementation, the more barriers at the socio-political context and organisation levels began to interfere with the process. Consequently, these barriers negatively affected factors at the user level (middle managers and employees).

#### Socio-political context level

##### Occurrence of external shocks

External shocks (i.e. COVID-19; Brexit; tight labour market) interfered negatively with the transition of HHR’s adoption to its implementation. These external shocks resulted in a stronger focus on the daily business and other competing priorities, whereby profit overruled the employees’ SE: *‘COVID-19 has brought many more things into focus. So if someone says, yes I would like to do a course and that costs so much, that is not going to happen, we are not going to make any costs’ (ID: 7).*

#### Organisation level

##### Challenges faced in the workplace

Internal shocks within the organisational setting also occurred. Due to budget cuts, supportive (financial) resources were not available anymore. *‘We as a company have been stripped so much to the bone that you have even less support when it comes to other things, projects’ (ID: 7).*

Staff turnover was another barrier for continuity: *‘(Name X) has fallen ill and is now out of service. In the third quarter of last year our (name Y) came along as interim HR, he promised a lot, but didn't deliver much and the support I needed for that. And now we have hired (name Z) and that is our new HR manager (….) due to all the staff changes we have been stuck for a while’ (ID: 4).*

Due to these barriers, the enthusiasm of the staff involved in HHR disappeared, which had been the basis for adoption. Additionally, senior management changes and their centralised decision-making process led to a new corporate vision and competing priorities on the business agenda; due to this, the employees’ SE was regarded as less important again. Middle managers themselves experienced a lack of decision-making authority to take action.

Other barriers were observed, such as a lack of practical resources in terms of time, room to execute HHR and overlapping HR initiatives, and hesitation continued: *‘As an organisation, we already have a lot of things that we already do (…) a lot of overlap. Also between the current projects and initiatives that we had already set up (…), we have doubts whether we should continue with the project’ (ID: 3).*

#### Intermediate user level – middle managers

##### The sandwich position

Due to the barriers at the organisation and socio-political context levels, middle managers felt placed in a difficult, dependent position. They experienced extra effort to regain support, lower energy levels and project fatigue. This resulted in a loss of support and enthusiasm. Often they had to rebuild the support of supervisors and employees. The middle managers of one organisation felt powerless when the senior management decided to terminate HHR. *‘It’s sometimes choosing your battles, and this is the choice, and we live up to it, it's that simple. Sometimes choices are made that make you happy and sometimes choices are made you feel less happy about’ (ID: 5).* Related to the feeling of powerlessness, frustration and disappointment were expressed: *‘The great disappointment has been for those people who invested time and energy again and then we are finally ready to use those tools in practice and then the entire project is cancelled…You try to communicate that nicely. Look, people are not stupid. And that is also my greatest frustration’ (ID 5).*

##### Perception about employees – creating an in- and out-group

The way the middle managers perceived their employees was a salient factor. They were prejudiced and characterised their employees as persons who struggle with language barriers, are difficult to reach, have reduced abstraction skills, have a different way of thinking, have low resilience and are a precarious group. Employees were considered as needing extra attention and support. Only a few middle managers described their employees as a vulnerable population and sought ways to give them a voice: *‘It's just looking at how you get the most active, how do you get the most out of their voice or own needs. I think that’s crucial and then it follows from this discussion that they need support or being taken by the hand. That seems to be important again’ (ID: 1).*

A lack of connection and interaction between middle managers and employees was observed. Gradually, an in- and out-group developed in terms of an ‘us-versus-them’ relationship at the organisational level. This seemed rooted in a lack of empathy and understanding, as middle managers who had once started in the low-skilled position of the employees were able to understand the employees better, showed empathy and did not experience an ‘us-versus-them’ relationship.

#### End-user level – employees

##### Perception of employees – ‘us-versus-them’ relationship

Employees themselves also expressed ‘us-versus-them’ thinking. Negative attitudes in terms of being sceptical and distrustful towards middle and senior management were observed*. ‘People are like a bit of staff versus management relationship, they are a bit sceptical about the line manager, like ‘nothing changes anyway’ (…) They’re a little suspicious, I think that's just part of it’ (ID: 9).* Additionally, a lack of social cohesion was experienced. *‘People are somehow a little scared of something, to say everything (…) that’s a shame’ (ID: 10).* The power, status and influence of significant others experienced by the employees played an important role in this regard.

#### Intervention level

##### SE regarded as easy to embrace but difficult to implement

HHR focuses on SE, and the interviewed middle managers described SE as a ‘container concept’ that was easily embraced at first, but difficulties arose when the concept had to be translated to the practice of their employees. *‘They can't make the nuance, just the word sustainable employability, they don't understand that. You have to make it easy and small. Almost children’s language’ (ID: 3).* Middle managers experienced a gap between their perceptions and those of their employees. The SE definition of the middle managers at the start predominated over the employees’ perceptions. This caused difficulties and a lack of skills to transition from adoption to implementation. *‘What bothers me, the moment I want to sell this project, I run into that it gets no real substance, because it’s such a container concept, it’s so extensive and you can have the feeling that you are very much involved with sustainability, while an employee is sitting next to you and does not experience it that way at all’ (ID: 7).* Moreover, their (HR) vision about SE and its importance did not seem to be congruent with that of others, such as direct supervisors who focused more on performance.

### Phase 3 implementation of HHR

Barriers at the socio-political context and organisation levels still affected both the middle managers and employees when the phase of implementation was finally reached for organisations 1 and 3.

#### Socio-political context and organisation level

##### Remaining shocks to the organisations

External and internal shocks remained present during the implementation phase, which interfered with the continuation of implementation. *‘I had everything ready and printed out everything from the toolbox (…) and it actually went quite well (…) and then COVID-19 came, and we could no longer stand together in a room’ (ID: 4).* Additionally, a lack of time to implement HHR properly was experienced due to daily job demands alongside the project of both the middle managers and employees: *‘The workload of the managers, who would facilitate it, is extremely high (ID: 2) (…) yes, but also employees, they are above their level and so much is currently asked of employees at the moment due to the circumstances (COVID-19)’ (ID: 1).*

##### Appearance of mental fatigue

During the implementation phase, middle managers still experienced the barriers at the organisation and socio-political context levels that were already experienced when transitioning to implementation, and middle managers behaved reactively. They felt dependent on what was happening in the wider system around them and again felt placed in a sandwich position. The daily job demands led to a lack of full focus, enthusiasm and involvement concerning HHR. *‘I had the feeling that it was a neglected child to me. Because you have high workloads and our reintegration processes always come first (…) So it came a little bit next to it, I don't feel like I gave it everything’ (ID: 1).*

Mental fatigue arose, because implementation took too long and required pushing and pulling. It seemed too demanding for the (HR) manager to invest in a dialogue, and thus implement HHR, with this lack of available resources. *‘Every time we started again, something is going on in the company. In the upcoming time, I’ll be busy with all the ongoing issues. I don't expect (Name X) either, as our HR department has been further stripped’ (ID: 4).*

#### End-user level – employees

##### The feeling of not being taken seriously and lack of communication

During implementation, employees expressed disappointment when they felt they were not being taken seriously. *‘Yes, I made that document and showed it to them. I did not find him (HR manager) very cooperative, because when I arrived, he was not there’ (ID: 9).* As a consequence, a negative attitude emerged, and enthusiasm eroded. The thought of ‘nothing happens anyway' already experienced in the transition phase was confirmed. Additionally, the lack of communication due to eroded enthusiasm suggested that the project was already over, *‘I actually thought it was all over, to be honest’ (ID: 8).*

#### Intervention level

##### Pleasant way of working, but no guarantee for success

HHR still represented a ‘pleasant way of working’ in the phase of implementation for the middle managers of organisation 1 and 3. At the same time, the implementation of HHR was experienced as a challenge due to the aforementioned factors at the user, organisation and socio-political context levels. 

### Overarching theme: steepness of the organisational hierarchy

Based on the factors reported by employees and middle managers to understand the variation in degree of adoption, transition from adoption to implementation, and then implementation, the overarching theme appeared to be a steeper organisational hierarchy. Table 5 shows the relationship between hierarchy and adoption, transition and implementation in the five organisations.

**Table 5:** The relationship between hierarchy and degree of adoption, transition and implementation and number of contact moments

	Steeper hierarchy			Flatter hierarchy	
Organisation	4	5	2	1	3
Phase					
1) Adoption	● (6)	◐ (4)	○ (7)	● (4)	● (4)
2) Transition adoption-implementation	◐ (13)	◐ (4)	○ (0)	◐ (2)	● (2)
3) Implementation	○ (0)	○ (0)	○ (0)	◐ (19)	◐ (10)

Note: 1) governmental institution, 2) cleaning company, 3) warehouse, 4) manufacturing company, and 5) meat-processing company

The degree: ● High; ◐ partial; ○ low

(#) = number of contact moments (internal (employer-employees) and external (researchers-organisations))

A steeper hierarchy was related to a lower degree of implementation (organisations 2, 4, 5) and defined as: the power of senior management to overrule subordinates (i.e. middle managers and employees) by not giving them a voice; and a lack of middle managers’ decision authority to push through and remediate the process to give employees more voice. The involved middle management layer had limited or no authority and seemed dependent on senior management for decision making. At the same time, these middle managers were dependent on immediate supervisors, who are closely involved with the employees. HHR cannot be implemented without the necessary support from other levels. These perceived power imbalances varied across organisations (being overruled was more prevalent in organisations 2 and 4, and a lack of authority by middle managers was more prevalent in organisations 2 and 5).

In contrast, a flatter hierarchy related to a higher degree of implementation (organisations 1 and 3), but was no guarantee for full implementation. Organisations 1 and 3 had a flatter hierarchy characterised by a power balance that prevented middle managers being overruled by senior management, and authority was exercised at the level of the middle managers, to give voice to the employees. In organisation 1, however, the lack of a power balance eventually emerged during the implementation phase as well, paralleled by partial implementation of HHR.

Simultaneously with the power imbalance processes, a social hierarchy emerged in all organisations, namely ‘us-versus-them’ thinking patterns. Different social norms were observed in terms of negative attitudes, the way of communication and behaviour among middle managers and employees. Middle managers spoke negatively about their employees and senior management, while employees felt distrustful towards their middle and senior management. These patterns proved to be harmful and reinforced the already existing power imbalances between senior and middle management and employees, hence the steepness of the organisational hierarchy.

## Discussion

This qualitative study analysed the process of adoption, transition from adoption to implementation, and implementation of the organisational intervention ‘Healthy HR’ (HHR) in five diverse organisations. All started with some degree of adoption, but only two out of five organisations implemented HHR partially; the other organisations did not achieve implementation. Employees and middle managers reported factors at all levels distinguished by Fleuren et al. [16]. The organisation and socio-political level factors came more into play after the adoption phase. The steepness of an organisation’s hierarchy appeared to be the overarching theme in understanding the degree of adoption, transition from adoption to implementation, and implementation. A steeper hierarchy constituted the main barrier.

All five organisations adopted HHR. SE, the core focus of the HHR intervention, was described as a container concept by the middle managers. This concept was useful for generating broad support and enthusiasm among stakeholders in the adoption phase. All agreed that SE was an important outcome, while concurrently having different perceptions about its meaning and translation. This empirical observation aligns with the diversity in conceptualisation and operationalisation of SE among different scholars [36]. In the implementation phase, this broad interpretation of SE lost its strength as it could not counterbalance the barriers. The broad concept of SE weakened the power among the middle managers.

Only two organisations fully transitioned from adoption to implementation and implemented HHR only to a certain extent. HHR builds on an egalitarian employer-employee dialogue and the willingness to give employees in low-skilled jobs more job control and voice. A mismatch occurred between this philosophy and the hierarchical organisational structures of the participating organisations. In line with Hadjisolomou and Simone [37], middle managers were caught between two structures, the social (i.e. social relations with employees) and organisational (i.e. power from senior management and supervisors). These experienced power structures resulted in a power imbalance, something which is also observed in other research [4, 38]. This power imbalance goes hand in hand with the observed us-versus-them (in-group versus out-group) thinking patterns among both management and the workfloor. According to social identity theory [39], the distinction between in- and out-groups is a social phenomenon and is described as ‘they (so the others) cannot speak our language’ [39]. The current findings show how difficult it is to change existing behavioural patterns in organisations and the behaviour of all stakeholders involved. The lower energy levels and negative attitudes among employees and middle managers affected the implementation and resulted in organisational cynicism, a common phenomenon in many organisations [40]. Hence, a steeper organisational hierarchy was related to worse outcomes (e.g. less satisfaction), a result that was found in previous research as well [25].

The context of COVID-19 amplified these processes even more. Organisations overburdened their employees and middle managers with high work demands and lost the bigger picture of the employees’ well-being [37]. This might have ultimately led to an increased distrust towards senior management and resistance to health initiatives [21]. Compromising the social cohesion might also have strengthened the ‘us-versus-them’ thinking patterns [40].

Both phenomena might be a result of a wider socio-political context [21]. From a neoliberal perspective, profit maximisation is the sole driver, which goes together with an increased emphasis on the individual responsibility of employees, thereby distracting attention from their health in the work environment. The perspective points to the distal influence of barriers at the macro-level, ultimately and negatively affecting the employee-employer relationship [41–43].

Closely related to this is the class discrimination that may underlie ‘us-versus-them’ thinking in organisations. This type of stigmatisation and stereotyping is very common and impacts organisational behaviour (i.e. high versus low educated), increasing the experience of inequality [44] and again pointing to macro-level forces influencing lower-level outcomes.

The longer the adoption and implementation phases lasted, the more the observed socio-political and organisational barriers evolved and started to interfere with them. The power at lower levels in organisations is too weak when the socio-political context and organisation barriers become more influential [4, 18, 45]. Such structural barriers grounded in socioeconomic and ideological systems are generally persistent [46]. Eventually, HHR was partially implemented at best, with flatter hierarchical organisations being more successful than steeper hierarchical organisations.

### Strengths and limitations

Three types of data were collected (data triangulation) in a set of five diverse organisations. By integrating these data types in the analyses, the researchers were able to follow and interpret the entire process in real-time. The QUAGOL approach to qualitative analysis strengthened the iterative process between different stages via constant interactive dialogue and data comparison with the members of the research team (disciplines in sociology; organisational psychology and occupational health) and made it possible to dive deeper into the research phenomenon [35].

Although five different organisations were studied, caution is recommended in transferring the findings to other organisations. One or two middle managers from each organisation were interviewed. However, not all organisations permitted the researchers to interview employees, primarily due to time constraints within the organisations and the problems resulting from COVID-19. Due to the small number of employees of one organisation (organisation 3), relevant perspectives might have been missed.

Moreover, a part of the data was collected during the first wave of the COVID-19 pandemic. Due to this, the interviews were collected through telephone or an online medium, which might have influenced the data collection through for example disturbance in internet connections and limited observations of non-verbal communication of the interviewees.Moreover, due to COVID-19, working procedures changed, and there was less interaction between middle managers and employees and among the employees. However, in the analysis, we did not perceive a lack of information – data triangulation might have counterbalanced infrequent flaws in online data collection. Further, content wise no clear relation between these changes and the lack of implementation was found. It might be, though, that the pandemic reinforced power differences and the ‘us-versus-them’ thinking patterns.

### Practical and future research implications

Although HHR was not successfully implemented, the findings add to existing knowledge on what does and what does not work, and for whom, when and under which circumstances regarding the implementation of organisational interventions [38, 47, 48]. With respect to the practical implications, To successfully create an organisational change, an adequate context analysis is needed to identify the organisation’s historical roots and its readiness for change [49]. For some organisations, HHR can be too disruptive and will not immediately match with existing organisational structures and cultures. When implementing an intervention, it is important to be aware of ‘path dependency’ (i.e. experiences and decisions made in the past) [50]. To yield success, new policies need to be developed that are in line with existing organisational institutions (policies, norms, cultures) [51], or the organisation needs to wait for an external force in the right direction, a so-called critical juncture [50] that cannot be created intentionally. A change in senior management might be an opportunity to put the right people in charge with a more democratic leadership style and a collective mindset, for whom hierarchy stands for accountability rather than for an autocratic leadership style [25].

From an institutional theory perspective, in organisations with a power imbalance and ‘us-versus-them’ thinking patterns, social norms need to be changed [52, 53]. For instance, an organisational culture of trust, respect, sincere interest and decentralised decision-making should be normalised before implementing an intervention like HHR. It may also be important to create awareness about stigmatising beliefs at the organisational level [44]. Opening a dialogue with the other group could be a way to transform the ‘us-versus-them’ thinking patterns to we-thinking [39] and provide more agency to the group of employees and reduce stigmatisation. These norms should be integrated into a democratic leadership style that promotes a true dialogue about what matters for the employees and that co-creates a culture of human dignity [41].

Furthermore, appointing fully focused ‘project champions’ (ambassadors of the project) could be helpful to increase the success. They should be able to translate the intervention into concrete actions and keep up the spirit, but they can only be effective in a culture when they have decision-making authority and are assertive enough to break through the power imbalances.

With respect to future research implications, the observed hierarchy seems more complex than the four levels of Fleuren et al. [16]. It is impossible to remove certain barriers, and hence the entire system should be addressed. Further research is needed on how to tackle or deal with these wider socio-political forces in occupational health research, which is impossible with the categorisation into four levels. For future implementation research and further development of HHR, the behavioral change wheel of Michie and colleagues could be a helpful framework to further analyses the context, the specific roles of different stakeholders and specify behavioral changes per target group (e.g. higher and middle managers and employees) [54]. Furthermore, researchers of future organisational interventions studies can learn from the presented persistent barriers involved in the adoption and implementation process of such interventions, act accordingly, and discuss them openly with organisations from the very beginning of the intervention process. Moreover, two groups were studied, the employees and the middle managers. Our findings indicated that the senior management, particularly in steeper hierarchical organisations, played an important role in the stagnation of the implementation process while we lacked direct interview materials from this group. Therefore, it would be better to involve them in future research and to increase the numbers of employees and middle managers as well.

## Conclusions

This qualitative study aimed to understand the degree of the adoption and implementation of the Healthy Human Resources (HHR) intervention aimed at improving the sustainable employability of employees in low-skilled jobs. Data triangulation was chosen to obtain a holistic understanding about the adoption and implementation process. The degree of adoption and implementation varies across the five organisations and was negatively affected by steeper hierarchies. Improving the sustainable employability of low-skilled employees thus appears difficult, as it requires breaking through deeply rooted power imbalances and pervasive ‘us-versus-them’ thinking patterns.

## Data Availability

The data that support the findings of this study are available on request from the corresponding author, EH. The data are not publicly available due to the personal and sensitive information from the involved organisations and their participants (employer representatives and employees). The data might be traced back to the organisations and individual respondents.

## Abbreviations

HHR: Healthy Human Resources
SE: Sustainable employability
HR: Human resource
QUAGOL: Qualitative Analysis Guide of Leuven
